# Cost-Effectiveness of Universal Routine Depression Screening for Adolescents in Primary Care

**DOI:** 10.1001/jamahealthforum.2025.0711

**Published:** 2025-05-02

**Authors:** Tran T. Doan, David W. Hutton, Davene R. Wright, Lisa A. Prosser

**Affiliations:** 1 Department of Health Systems, Management, and Policy, Colorado School of Public Health, Aurora; 2 Department of Health Management and Policy, University of Michigan School of Public Health, Ann Arbor; 3 Department of Population Medicine, Harvard Pilgrim Health Care Institute and Harvard Medical School, Boston, Massachusetts; 4 Susan B. Meister Child Health Evaluation and Research Center, Department of Pediatrics, University of Michigan Medical School, Ann Arbor

## Abstract

**Importance:**

Approximately one-fifth of adolescents in the US experience a major depressive episode each year. Universal depression screening for adolescents is recommended as part of routine pediatric primary care, but its cost-effectiveness is unclear.

**Objective:**

To evaluate the cost-effectiveness of universal routine depression screening in adolescent primary care compared with usual care.

**Design, Setting, and Participants:**

This economic evaluation used a decision-analytic model with an embedded state-transition submodel and annual transitions. A hypothetical population of 1000 adolescents and young adults from ages 12 to 22 years, including 12 demographic groups of disaggregated combinations of sex (female and male) and race or ethnicity (American Indian or Alaska Native; Asian, Native Hawaiian, or Pacific Islander; Black or African American; Hispanic, Latino, or Spanish; White; and multiracial or other race or ethnicity) was simulated in pediatric primary care settings.

**Exposures:**

Universal depression screening of varying frequencies, including annual, biennial, and single-time screening at age 12 years, compared with usual care, defined as 20% annual screening rate.

**Main Outcomes and Measures:**

Costs, health effects as measured by quality-adjusted life-years (QALYs) and depression-free days, and incremental cost-effectiveness ratios (ICERs) from the health care sector and limited societal perspectives.

**Results:**

A universal annual screening policy had an ICER of $66 822 per QALY or $84 per depression-free day gained compared with single-time screening from the limited societal perspective, including caregiver time costs. Universal single-time screening had an ICER of $44 483 per QALY and $62 per depression-free day gained compared with usual care. Targeted universal depression screening was more cost-effective for female individuals and those who identified as Hispanic, Latina, or Spanish, multiracial, or other race or ethnicity. Results were sensitive to treatment recovery rates, depression health state utility scores, treatment costs involving psychotherapy, suicide-related hospitalization costs, and initial depression prevalence at age 12 years. In approximately 99.8% of probabilistic simulations, universal annual screening had an ICER less than $150 000 per QALY threshold.

**Conclusions and Relevance:**

The study results suggest that universal annual depression screening for adolescents in primary care is cost-effective compared with a $100 000 per QALY willingness-to-pay threshold. Universal annual screening may be more cost-effective if health systems invest in efforts to enhance family access to telemedicine behavioral health, decrease treatment costs, or improve treatment effectiveness. Future analyses could examine whether additional potentially associated demographic factors, such as gender orientation, sexual identity, rurality, or comorbidities, affect cost-effectiveness outcomes.

## Key Points

**Question:**

What is the cost-effectiveness of universal routine depression screening for adolescents and young adults as performed in pediatric primary care settings compared with usual care?

**Findings:**

In this economic evaluation using a decision-analytic model, universal annual depression screening had an incremental cost-effectiveness ratio of $66 822 per quality-adjusted life-year gained compared with single-time screening for a hypothetical population of 1000 adolescents from a limited societal perspective. Single-time screening had an incremental cost-effectiveness ratio of $44 483 compared with usual care.

**Meaning:**

The study results suggest that universal annual screening is a cost-effective option and could be more cost-effective if health systems invest in efforts to enhance family access to telemedicine behavioral health, decrease treatment costs, or enhance treatment effects.

## Teaser

This economic evaluation examines the cost-effectiveness of universal routine depression screening in adolescent primary care compared with usual care.

## Introduction

Universal routine screening of major depressive disorder (depression) is often recommended for adolescents in primary care.^1,2,3^ Approximately 20% of adolescents in the US experienced depressive symptoms in 2019,^4^ yet adolescent depression is underdiagnosed^1,5^ and undertreated.^6,7^ Adolescents with depression are more likely to use illicit substances, experience anxiety or eating disorders, and experience suicidality.^8,9^ Depression disproportionately affects adolescents who identify as female, Latinx, or multiracial.^10,11^ Adolescent depressive symptoms, even when mild, are associated with increased health care utilization and costs.^12^

Three national organizations jointly declared a national state of emergency in children’s mental health in 2021^13^ and recommended universal adolescent depression screening of varying frequency and provided the availability of systems in place to ensure accurate diagnosis, effective treatment, and appropriate follow-up.^2,5,10^ Given a shortage of mental health specialists,^14^ pediatricians and other primary care physicians can play a supportive role in screening and initiating treatment for adolescent depression.^5,15,16^ A school-based trial found that universally screened adolescents have double the likelihood of treatment initiation.^17,18^ In contrast to robust evidence supporting depression screening in adults,^19^ there is arguably insufficient evidence for screening in adolescents.^1^ National bodies recommending adolescent depression screening are based on indirect evidence rather than direct evidence for screening benefits and harms.^2,5,10,20^ The American Academy of Pediatrics (AAP) argued that the lack of trials on screening efficacy “becomes less relevant”^1^ considering the mounting evidence on screening validity, feasibility of screening and treatment implementation, and treatment efficacy.^1,5^

Conducting a clinical trial to establish screening efficacy on adolescent health outcomes presents challenges. In primary care contexts in which universal depression screening is already occurring, withholding screening could be unethical. Multiple system-level barriers must be addressed for screening to be efficacious; screened patients must also receive an accurate diagnosis and timely treatment. Priorities may be given to funding treatment-based trials vs screening-based ones. Considering these challenges, a simulation model provides a timely, lower-cost alternative to clinical trials for testing alternative hypothetical scenarios of health systems improvement from screening implementation to treatment uptake and by conducting uncertainty analyses to test the strength of these scenarios in terms of changing long-term outcomes.

A cost-effectiveness model may simulate the comparative effectiveness and costs of universal adolescent depression screening over the long term. This study aimed to measure the costs, health effects, and cost-effectiveness of universal routine depression screening for adolescents in primary care over 10 years compared with usual care.

## Methods

### Approach

A decision-analytic model was developed that projected outcomes for a hypothetical US adolescent and young adult cohort at an initial age of 12 years using annual cycles over adolescence (a 10-year horizon). This analysis followed conduct and reporting guidelines by the Second Panel on Cost-Effectiveness in Health and Medicine^21,22^ (Table 1^8,23,24,25,26,27,28,29,30,31,32,33,34,35,36,37,38,39,40,41,42,43,44,45,46,47,48,49,50,51,52^) and Consolidated Health Economic Evaluation Reporting Standards (CHEERS) reporting guideline (Table 2), respectively.^53^ This study was considered exempt from human participants review by the institutional review board of the University of Michigan.

**Table 1.** Model Inputs for Cost-Effectiveness Analysis

Input	Base case estimate	Lower estimate	Upper estimate	Source
Event probabilities^a^				
Depression-free to depression from ages 12-13 y	0.082	0.062	0.102	Doan et al,^23^ 2024; Harris et al,^24^ 2009
Depression-free to depression from ages 13-14 y	0.055	0.035	0.075	Doan et al,^23^ 2024; Harris et al,^24^ 2009
Depression-free to depression from ages 14-15, 15-16, and 16-17 y	0.020	0.010	0.030	Doan et al,^23^ 2024; Harris et al,^24^ 2009
Depression to recovery from ages 17-18 y and older age intervals	0.010	0.005	0.015	Doan et al,^23^ 2024; Harris et al,^24^ 2009
Depression to recovery from ages 12-13 y	0.287	0.187	0.387	Doan et al,^23^ 2024; Harris et al,^24^ 2009
Depression to recovery from ages 13-14 y	0.164	0.064	0.264	Doan et al,^23^ 2024; Harris et al,^24^ 2009
Depression to recovery from ages 14-15, 15-16, and 16-17 y	0.064	0.044	0.084	Doan et al,^23^ 2024; Harris et al,^24^ 2009
Depression to recovery from ages 17-18 y and older age intervals	0.094	0.074	0.114	Doan et al,^23^ 2024; Harris et al,^24^ 2009
Recovery to depression (relapse) from ages 12-13 y	0.305	0.205	0.405	Doan et al,^23^ 2024; Harris et al,^24^ 2009
Recovery to depression (relapse) from ages 13-14 y	0.200	0.100	0.300	Doan et al,^23^ 2024; Harris et al,^24^ 2009
Recovery to depression (relapse) from ages 14-15, 15-16, and 16-17 y	0.098	0.078	0.118	Doan et al,^23^ 2024; Harris et al,^24^ 2009
Recovery to depression (relapse) from ages 17-18 y and older age intervals	0.045	0.025	0.065	Doan et al,^23^ 2024; Harris et al,^24^ 2009
Relative risk of suicidality with depression^b^	2	1.5	2.5	Riera-Serra et al,^25^ 2023
US depression prevalence at age 12 y^c^	0.083	0.011	0.266	National Survey on Drug Use and Health 2019^8^
US recovery prevalence at age 12 y^d^	0.02	0	0.05	Assumption^d^
US treatment prevalence at age 12 y	0.1	0	0.33	Rodgers et al,^26^ 2022; Flores et al,^6^ 2023; Merikangas et al,^27^ 2011
US mortality rates	Varies	–	–	US Centers for Disease Control and Prevention WONDER database^28^
Direct costs, $				
Screening^e^	4.74	3.92	6.40	CMS physician fee schedule 2023^29^
Physician examination for new patients^f^	167.40	152.93	215.51	CMS physician fee schedule 2023^29^
Physician examination for established patients^g^	128.43	117.41	164.94	CMS physician fee schedule 2023^29^
Suicide attempt	9000	8000	35 000	2010 Washington Healthy Youth Survey^30^; Perkins et al,^31^2005; Shepard et al,^32^ 2016
Died by suicide	5000	3550	9000	2010 Washington Healthy Youth Survey^30^; Shepard et al,^32^ 2016
Antidepressant medication^h^	149.52	NA	NA	Veteran Affairs federal supply schedule 2023^33^
Psychotherapy per session^i^	147.07	140.96	205.30	CMS physician fee schedule 2023^29^
Psychotherapy sessions per year	20	10	40	Assumption; expert opinion
Indirect time costs, $				
Adult mean hourly wage	33.82	15.00	40.00	US Bureau of Labor Statistics 2023^34^
Caregiver time, h^j^	1.5	1	3	Assumption
Care access probabilities				
Well-child care visits^k^	0.576	0.486	0.7	Medicaid and CHIP scorecard 2023^35^
Probability of screening				
Usual care	0.2	0.1	0.45	Sekhar et al,^36^ 2019; Bose et al,^37^ 2021; Lewandowski et al,^38^ 2016; Zenlea et al,^39^ 2014; expert opinion
Universal screening	0.9	0.8	1	Bose et al,^37^ 2021; MN Community Measurement^40^; expert opinion
Probability of treatment^l^				
Given no screening	0.05	0	0.1	Expert opinion
Given true-positive screen result	0.90	0.47	1	Expert opinion
Given false-positive screen result	0.05	0	0.1	Expert opinion
Given a negative screen result	0	NA	NA	Expert opinion
Under usual care	0.33	0.1	0.47	National Survey on Drug Use and Health 2019^8^; Rodgers et al,^26^ 2022; Flores et al,^6^ 2023; Merikangas et al,^27^ 2011; expert opinion
PHQ-9 sensitivity (true positive)	0.895	0.8	1	Richardson et al,^41^ 2010
PHQ-9 specificity (true negative)	0.775	0.7	1	Richardson et al,^41^ 2010
Probability of suicide attempts resulting in hospitalization^m^	0.0875	0.055	0.125	Gaylor,^42^ 2023
Discounting rate	0.03	0	0.09	Assumption
Health effectiveness				
Utility score				
Depression free	0.98	0.95	1	Ward et al,^43^ 2023
Depression	0.542	0.44	0.67	Mann et al,^44^ 2009; Sapin et al,^45^ 2004
Recovery	0.85	0.825	0.875	Mann et al,^44^ 2009; Sapin et al,^45^ 2004
Death	0	NA	NA	Assumption
Depression-free d				
Depression free	335	300	365	Assumption
Depression^n^	96	74	117	Domino et al,^46^ 2008
Recovery^o^	196	174	217	Domino et al,^46^ 2008
Treatment effectiveness^p^				
Relative risk of relapse (recovery to depression)^q^	0.61	0.5	0.8	Emslie et al,^47^ 2008
Relative risk of depression-free d	1.2	1	2	Lynch et al,^48^ 2009; Domino et al,^46^ 2008
Medication and psychotherapy combination				
Relative risk of recovery (depression to remission)	2	1.5	2.5	Kennard et al,^49^ 2006
Relative risk of suicidality (continue to have depression)^r^	0.79	0.6	1	March et al,^50^ 2004; Vitiello et al,^51^ 2009
Probability of discontinuing treatment^s^	0.36	0.26	0.46	March et al,^52^ 2007
Antidepressant medication only				
Relative risk of recovery (depression to remission)	1.35	1	1.5	Kennard et al,^49^ 2006
Relative risk of suicidality (continue to have depression)^t^	1.37	1	1.5	March et al,^50^ 2004; Vitiello et al,^51^ 2009
Probability of discontinuing treatment^s^	0.50	0.4	0.6	March et al,^52^ 2007
Psychotherapy only				
Relative risk of recovery (depression to remission)	1.1	0.9	1.5	Kennard et al,^49^ 2006
Relative risk of suicidality (continue to have depression)^r^	0.59	0.5	1	March et al,^50^ 2004; Vitiello et al,^51^ 2009
Probability of discontinuing treatment^s^	0.50	0.4	0.6	March et al,^52^ 2007

Abbreviations: CHIP, Children’s Health Insurance Program; CMS, US Centers for Medicare & Medicaid; *CPT*, *Current Procedural Terminology*; NA, not applicable; PHQ-9, Patient Health Questionnaire 9-Item; TADS, Treatment for Adolescents With Depression Study.

^a^ Transition probabilities for a general US adolescent cohort were estimated by applying a survival analysis on data from the National Longitudinal Study from Adolescent to Adult Health from waves 1 to 4 and calibrated to data from the 2019 National Survey on Drug Use and Health. For transition probabilities across sex, race and ethnicity, and sex and race and ethnicity combinations.

^b^ For individuals with depression in the model, we multiplied the relative risk of suicidality with having depression and the annual probability of suicide attempts or the annual probability of death by suicide.

^c^ We derived the lower estimate for US depression prevalence at age 12 years from the prevalence rate for male American Indian or Alaska Native individuals and the higher estimate from female individuals of multiracial or other racial or ethnic backgrounds.

^d^ To our knowledge, the estimate for the US age-specific recovery prevalence for youth at age 12 years cannot be found in the literature. We assumed that a 2% (range, 0%-5%) age-specific recovery prevalence estimate was reasonable, considering that the age-specific depression prevalence at age 12 years was 8.3%.

^e^ We derived base case estimates from applying *CPT* code 96127. Lower and higher estimates came from the range in the CMS physician fee schedule lookup tool.

^f^ We derived the base case estimate from applying the *CPT* code 99204, lower estimate from 99203, and higher estimate from 99205.

^g^ We derived the base case estimate from applying the *CPT* code 99214, lower estimate from 99213, and higher estimate from 99215.

^h^ We determined the cost of a fluoxetine hydrochloride, 20 mg, tablet per day for 12 months.

^i^ We derived the base case estimate came from applying the *CPT* code 90837.

^j^ We assumed a parent or caregiver accompanied the patient 100% of the time.

^k^ We derived the base case estimate from mean estimates for commercial health maintenance organizations and lower bound from mean estimates for Medicaid health maintenance organizations and assumed the higher estimate.

^l^ For individuals who had not received treatment and had depression at the beginning of the model, we applied an annual probability of receiving treatment.

^m^ We derived the base case estimate from calculating the weighted averages of suicide attempt prevalence in female and male participants aged 14 to 18 years in the 2019 US Youth Risk Behavior Survey. We derived the lower estimate from the lower bound of the 95% CI for male individuals. We derived the higher estimate from the higher bound of the 95% CI for female individuals.

^n^ Using data from the TADS randomized clinical trial, there were an estimated 22 depression-free days among individuals who had depression during 84 days in the trial. We extrapolated this estimate to 365 days and applied a wide uncertainty range for sensitivity analysis.

^o^ Using data from TADS, there were an estimated 45.1 depression-free days among individuals who achieved remission during 84 days in the trial. We extrapolated this estimate to 365 days and applied a wide uncertainty range for sensitivity analysis.

^p^ Treatment effectiveness came from the 2004 TADS.

^q^ Relapse rates were reported for youth participants aged 7 to 18 years who were assigned to the fluoxetine medication treatment group or placebo group. In the model, we assumed that the relative risk of relapse rates was the same for individuals who were treated for depression regardless of treatment type (fluoxetine medication only, psychotherapy only, or a combination therapy of both). We also applied a wide uncertainty range for the sensitivity analysis.

^r^ We applied the relative risk of suicidality due to depression treatment on an annual basis.

^s^ The probability of discontinuing treatment was applied once after the first year of treatment initiation.

^t^ We applied the relative risk of suicidality due to depression treatment with medication only during the first year and then no increased risk in subsequent years.

**Table 2.** Projected Total and Incremental Costs, Depression-Free Days, and Quality-Adjusted Life-Years (QALYs) per 1000 US Adolescents Young Adults From Ages 12 to 22 Years

Strategy	2023 $								Total depression free d (thousands)	Incremental depression-free d gained (thousands)	Total QALYs	Incremental QALYs gained
	Universal screening costs (thousands)		Treatment costs (thousands)		Total costs (thousands)		Incremental total costs (thousands)					
	Health care sector	Limited societal^a^	Health care sector	Limited societal^a^	Health care sector	Limited societal^a^	Health care sector	Limited societal^a^				
Usual care^b^	132	181	1893	1995	9687	9789	NA	NA	2475	NA	7766	NA
Single-time screening	197	264	3001	3154	10 669	10 821	$982	1032	2492	16.5	7789	23.2
Biennial screening	281	384	3934	4148	11 516	11 730	847	909	2502	9.9	7800	10.7
Annual screening	542	740	5309	5652	12 765	13 108	1249	1378	2519	17.4	7824	23.6

Abbreviation: NA, not applicable.

^a^ Limited societal perspective included time cost.

^b^ Usual care was defined as a 20% annual screening rate and 33% treatment initiation rate.

### Interventions

Four universal depression screening options were modeled for adolescents in primary care: (1) annual screening following AAP guidelines; (2) biennial screening; (3) single-time screening at age 12 years; and (4) usual care, which was defined as a 20% annual screening rate^36,37,38,39^ and 33% treatment rate.^6,8,26,27^ For the base case, annual and biennial screenings assumed an enhanced screening rate (90%) with wide uncertainty ranges (Table 1). For single-time screening, enhanced screening (90%) was applied for the first year and then usual care screening (20%) was applied for remaining years. Annual, biennial, and single-time screenings also assumed an enhanced treatment rate (90%).^17,37^ For all screening options, 58% of adolescents had an annual well-child visit.^35^ These health care utilization assumptions were tested in uncertainty analyses.

### Model Structure

**Figure 1.**
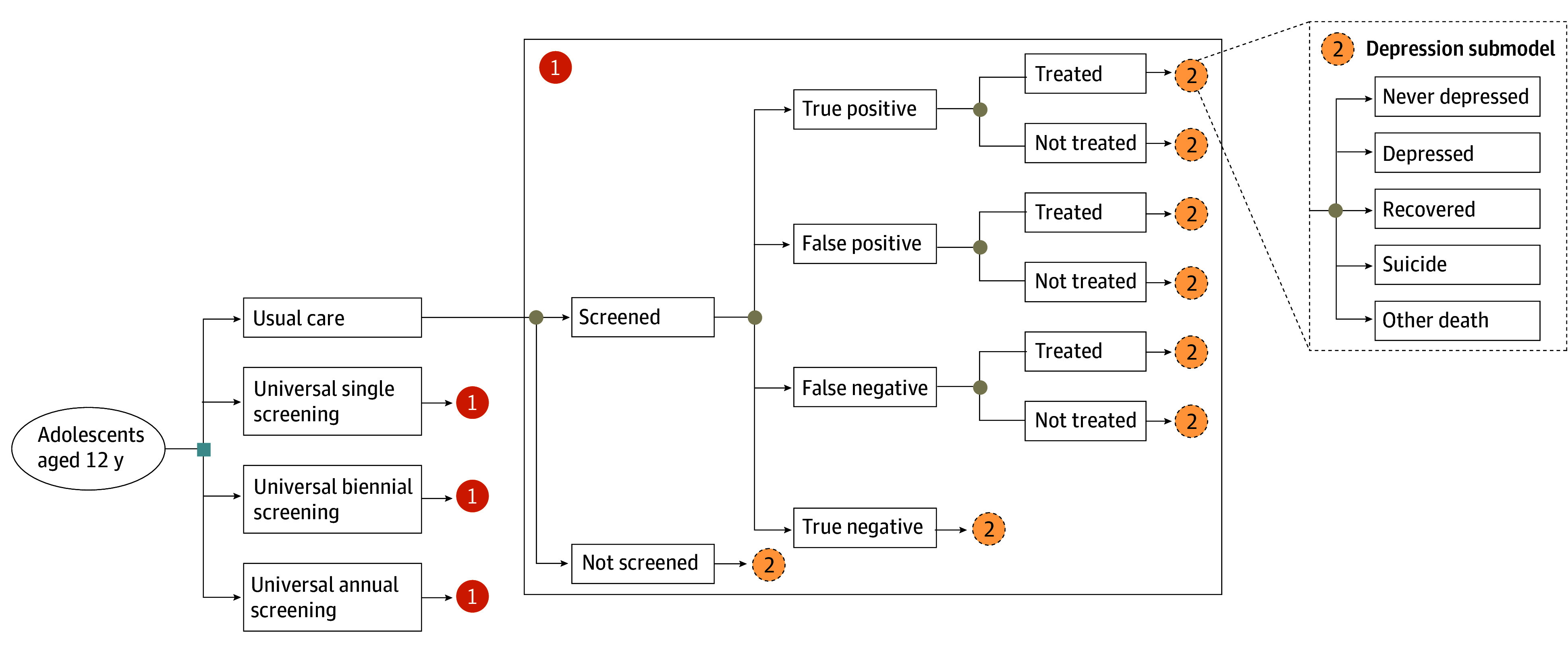
Decision-Analytic Tree Model for Universal Routine Screening of Major Depression for Adolescents in Primary Care Settings

The decision-analytic model included probabilities of a well-child visit, screening, diagnosis, and treatment (Figure 1). Individuals were determined to have depression if they screened a cutoff score of 11 or higher on the Patient Health Questionnaire 9-Item (PHQ-9),^41^ as indicated by the AAP.^5^ The PHQ-9 alone is not a diagnostic tool, but its sensitivity and specificity can serve as conservative lower estimates of diagnostic accuracy when supplemented by clinician interviews. The model was programmed in TreeAge Pro, version 2022.

The model included a depression submodel^23^ (Figure 1) composed of 6 health states following screening. Individuals who initially did not have depression could potentially transition to depression or death. Individuals who initially had depression could potentially transition to recovery or death. Individuals with recovery could potentially experience a relapse back to depression or transition to death. Individuals with depression or recovered individuals could be treated.

### Model Inputs and Data Sources

Model inputs included event probabilities, costs, health effects, and treatment effectiveness (Table 1). Expert opinion was also used to confirm model structures. Expert panelists comprised 3 leading US adolescent mental health physicians from the University of Washington, University of Wisconsin, and University of Pittsburgh.

#### Event Probabilities

We estimated transition probabilities using data from the National Longitudinal Study of Adolescent to Adult Health (Add Health)^24,54^ and the 2019 National Survey on Drug Use and Health,^8^ as described previously.^23^ Transition probabilities were estimated for 12 demographic groups of combinations of sex (female and male) and race or ethnicity (American Indian or Alaska Native; Asian, Native Hawaiian, or Pacific Islander; Black or African American; Hispanic, Latino, or Spanish; White; and multiracial or other race or ethnicity [individuals not identifying as aforementioned categories]). Race or ethnicity was self-reported.

#### Costs

We included direct costs for outpatient visits, hospitalizations, and treatment (Table 1). The base case analysis assumed annual costs of combination treatment and 2 follow-up physician visits. For the limited societal costing perspective, additional indirect costs of lost adult wages were included for caregiver time spent traveling and accompanying adolescents on physician visits. Costs were inflated to 2023 USD using the gross domestic product implicit price deflator.^55^

#### Health-Related Quality of Life Adjustments

We derived health utilities for depression-related states from peer-reviewed literature^44,45^ using the EuroQoL (EQ-5D), a generic preference-based survey instrument that estimates health-related quality of life.^56^ Health utility represents a quantitative scale from 0 (death) to 1 (perfect health).^21^ Quality-adjusted life-years (QALYs) were calculated by multiplying state-specific utilities and time in years.

#### Treatment Effectiveness

The base case applied the recommended combination treatment of a first-line antidepressant (fluoxetine) and psychotherapy.^5,50^ Treatment effectiveness involved applying risk ratios for relapse, remission, and suicidal attempts. Treatment effectiveness came from the Treatment for Adolescent with Depression Study,^50^ a multicentered clinical trial that randomized 439 adolescents to receive 12 weeks of treatment with fluoxetine medication, psychotherapy, their combination, or a pill placebo. Treatment groups were followed-up to 5 years to evaluate relapse, remission, and suicidality over time.

### Base Case Analysis

Outcomes included costs, health effects (including QALYs and depression-free days), and incremental cost-effectiveness ratios (ICERs), which were defined as the difference in costs between 1 intervention and the next best, nondominated intervention divided by the difference in QALYs.^22^ An intervention was determined dominated if there was poorer health at a higher cost compared with alternatives.^22^ Analyses were conducted from the health care sector and limited societal perspective, including caregiver time costs accompanying physician visits. Future outcomes were discounted 3% annually.

### Statistical Analysis

#### Sensitivity Analyses

Deterministic and probabilistic sensitivity analyses were conducted on reasonable ranges of input values (Table 1). In the probabilistic sensitivity analysis, a second-order Monte Carlo simulation was conducted using 10 000 iterations. β Probability distributions were assigned to transition probabilities, γ distributions to costs and utilities, and log-normal distributions to treatment effectiveness risk ratios.

#### Scenario Analyses

Scenario analyses were performed to study heterogeneity in the adolescent cohort, treatment effects, and enhanced health care utilization. We also varied discount rates (1.5%-5%) and time horizons (3-5 years), added caregiver time costs to in-person psychotherapy, and added adolescent time costs to clinical and psychotherapy.

To study heterogeneity, the adolescent cohort was varied by disaggregated sex and race or ethnicity combinations by modifying the following inputs: (1) initial depression prevalence, (2) age-specific mortality, and (3) transition probabilities for each demographic group. To study treatment effects, treatment type (medication only or psychotherapy only) was varied by modifying the following inputs: (1) treatment costs, (2) recovery risk ratios, and (3) suicidality risk ratios. We evaluated the scenarios of removing treatment discontinuation (applied once after the first year of treatment initiation) and adding treatment reinitiation after discontinuation. To study enhanced health care utilization, we evaluated scenarios of enhanced level of universal screening accompanied by (1) status quo well-child visit and status quo treatment, (2) enhanced well-child visit and status quo treatment, or (3) enhanced well-child visit and enhanced treatment compared with usual care.

## Results

### Health Outcomes

Usual care yielded 2 475 434 depression-free days for a hypothetical US cohort of 1000 adolescents and young adults aged 12 to 22 years (Table 2). Compared with usual care, universal single-time screening added 16 539 depression-free days, biennial screening added 26 399, and annual screening added 43 815 during the same period. Also compared with usual care, a cumulative gain of 23.2 QALYs was projected for single-time screening, 33.9 QALYs for biennial screening, and 57.4 QALYs for annual screening (Table 2).

### Costs

From the health care sector perspective, total costs per 1000 adolescents were $9.7 million for usual care, $10.7 million for single-time screening, $11.5 million for biennial screening, and $12.8 million for annual screening (Table 2). From the societal perspective, total costs were $9.8 million for usual care, $10.8 million for single-time screening, $11.7 million for biennial screening, and $13.1 million for annual screening. Modeling the entire US population size of adolescents aged 12 years in 2023 (approximately 4.1 million),^28^ the societal costs would total $40.1 billion, $44.4 billion, and $53.7 billion, respectively.

### Base Case Analysis

From the health care sector perspective, ICERs were $42 317 per QALY and $59 per depression-free day gained for universal single-time vs usual care screening and $61 240 per QALY and $77 per depression-free day gained for annual vs single-time screening (Table 3). From the societal perspective, ICERs were higher at $44 483 per QALY and $62 per depression-free day for single-time vs usual care screening and $66 822 per QALY and $84 per depression-free day for annual vs single-time screening. Universal biennial screening was dominated by extended dominance between single-time screening and annual screening, meaning biennial screening produced lower health benefits than annual screening at a higher ICER.^22^

**Table 3.** Base Case Analysis Showing Incremental Cost-Effectiveness Ratios Among 1000 US Adolescents From Age 12 to 22 Years

Strategy	2023 $			
	Health care sector perspective		Limited societal perspective^a^	
	Cost per QALY gained	Cost per depression-free d gained	Cost per QALY gained	Cost per depression-free d gained
Usual care^b^	NA	NA	NA	NA
Single-time screening compared to usual care	$42 317	$59	$44 483	$62
Biennial screening compared to single-time screening	$79 514^c^	$86^c^	$85 299^c^	$92^c^
Annual screening compared to single-time screening	$61 240	$77	$66 822	$84
Annual screening compared to usual care	$53 593	$72	$57 795	$76

Abbreviations: NA, not applicable; QALYs, quality-adjusted life-years.

^a^ Limited societal perspective included time cost.

^b^ Usual care was defined as a 20% annual screening rate and 33% treatment initiation rate.

^c^ Dominated by extended dominance between single-time screening and annual screening in that it achieved fewer health gains compared with annual screening (see Table 2) at a higher incremental cost-effectiveness ratio.

### Sensitivity Analyses

Model inputs that most affected ICERs were the recovery risk ratio, depression utility score, treatment costs (ie, hours spent in psychotherapy and per-session psychotherapy cost), suicide-related hospitalization costs, and starting depression prevalence (eFigure 2 in Supplement 1). ICERs remained less than $84 000 for single-time vs usual care screening and $118 000 for annual vs single-time screening across all sensitivity analyses (eTable 3 in Supplement 1).

**Figure 2.**
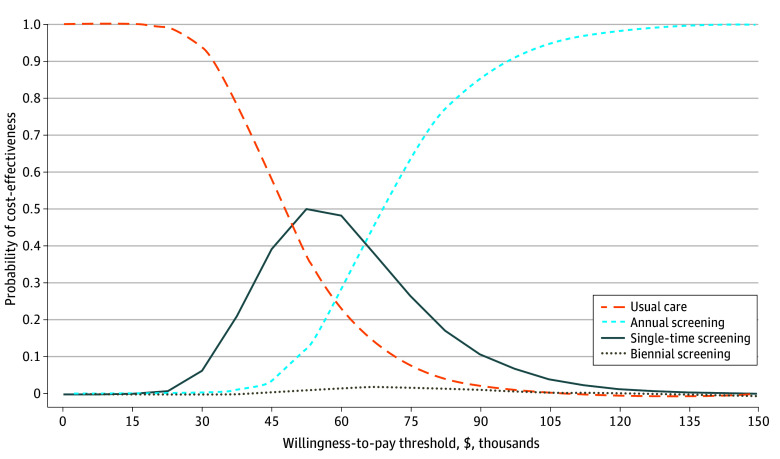
Cost-Effectiveness Acceptability Curve of Universal Routine Depression Screening Options Compared With Usual Care

A probabilistic sensitivity analysis comparing universal screening options showed that annual screening was the preferred option in 99.8% of 10 000 simulations at a $150 000 per QALY willingness-to-pay threshold. At lower thresholds, multiple screening policies demonstrated cost-effectiveness except biennial screening. For example, at less than $50 000 per QALY, usual care was most likely preferred (Figure 2). At approximately $52 500 per QALY, single-time screening was most likely preferred, followed by usual care and annual screening. At greater than $67 500 per QALY, annual screening was most likely preferred.

### Scenario Analyses

#### Disaggregated Sex and Race or Ethnicity Combinations

Across all demographic groups, universal annual screening was most cost-effective among female individuals who identified as multiracial, other race or ethnicity, or Hispanic, Latina, or Spanish (eTable 4 in Supplement 1). Annual screening was less cost-effective among male individuals who identified as American Indian or Alaska Native or Black or African American who had a low initial depression prevalence.

#### Alternate Treatment Options

When treatment was psychotherapy only, ICERs increased to $188 470 per QALY for single-time vs usual care screening and $340 172 per QALY for annual vs single-time screening (eTable 5 in Supplement 1). When treatment was medication only, the ICERs decreased to $21 447 per QALY and $50 150 per QALY, respectively. Psychotherapy only without medication might be less favorable due to long remission timelines and costs compared with medication-only treatment.^50,57^ Medication only demonstrated higher risk of suicidality and associated outcomes than other treatments, although increased suicidality risk from using medication typically occurred during the first 6 months or less, and ongoing management was recommended.^51^ Still, combination treatment is the AAP-recommended course.^7^

#### Enhanced Health Care Utilization

Compared with the base case, ICERs were less favorable in scenarios with treatment at the status quo rate ($49 275-$82 184/QALY), even when combined with increased well-child visits ($48 915-$84 377/QALY) (eTable 5 in Supplement 1). Enhancing rates of well-child visits and treatment utilization yielded slightly more favorable ICER for single-time vs usual care screening ($42 551/QALY) and slightly less favorable ICER for annual vs single-time screening ($69 953/QALY).

#### Additional Scenario Analyses

If caregiver time costs were added to in-person psychotherapy (vs telehealth), then ICERs became less favorable to $58 095 per QALY for single-time screening and $84 264 per QALY for annual screening (eTable 5 in Supplement 1). Adding adolescent time costs also yielded less favorable ICERs ($46 893-$70 512/QALY). ICERs did not differ much when removing treatment discontinuation ($45 638-$66 592 per QALY) or adding treatment reinitiation after discontinuation ($45 292-$66 414 per QALY). Varying discount rates from 1.5% to 5% yielded minimal changes ($43 726-$68 869 per QALY). Reducing the time horizon from 10 to 3 years increased ICERs ($57 806-$157 591 per QALY).

## Discussion

In this economic evaluation, universal annual screening for adolescent depression yielded a higher cost-effectiveness ratio than single-time screening but both were cost-effective strategies under common willingness-to-pay thresholds. This study provides the most updated estimate for the cost-effectiveness of a primary care adolescent universal depression screening program and identifies factors, like treatment rates, costs, and effectiveness, that affect the cost-effectiveness of screening.

Our analysis, disaggregated by demographic combinations of sex and race or ethnicity, suggests that adolescent female individuals from multiracial, other race or ethnicity, or Hispanic, Latina, or Spanish backgrounds could benefit the most from universal routine screening in primary care and especially if coupled with enhanced treatment uptake.^6,9,10,26,58^ A school-based trial found that universal adolescent depression screening was associated with improved treatment referrals but not reduced racial or ethnic disparities in these referrals.^59^ However, this trial tested single-time screening and not routine screening, which may present additional opportunities to reduce disparities through follow-up visits and care coordination.^5,16^ There remain urgent needs for health systems and policy reforms to close widening disparity gaps in mental health access, especially among Black and Hispanic children,^26^ rural children,^60^ and low-income families.^60^ Therefore, it is important to promote engagement with diverse families in primary mental health systems beyond the delivery points of diagnosis and treatment referral.^4,59,61^

Universal adolescent depression screening may be controversial. Critics argue that universal depression screening may lead to unintentional harm, including overdiagnosis, overtreatment, and wasted costs.^62,63,64,65^ In pediatric primary care, implementation studies showed that universal depression screening was deemed feasible and acceptable to families^66^ and was associated with increased early identification and treatment.^37,61^ Caregivers are more likely to consent to treatment if referrals come from universal systematic screening vs screening-triggered outcomes.^16,59^ To our knowledge, there are no causal studies demonstrating the efficacy of universal adolescent depression screening in primary care specifically.^1,63^ However, 3 school-based trials tested screening efficacy and found associations between universal adolescent depression screening and improved problem identification, treatment referrals, and treatment initiation.^17,59,67^ While prior studies examined screening effectiveness on a single-time basis,^17,37,59,67^ our study provides evidence that screening on a routine annual basis could be a cost-effective measure for implementing.

One simulation study evaluated the comparative effectiveness, but not cost-effectiveness, of universal routine depression screening in adolescent primary care and concluded that screening achieved relatively small effects (<5% of achievable effect) on population mental health.^4^ Our study found that universal annual screening added 44 depression-free days (<2% improvement) per child over 10 years compared with usual care. While these study outcomes are not directly comparable, these modest health effects may result in multiple systems failures along the pediatric-mental delivery care continuum (eg, low access to care).^4^ Our findings suggest that sustaining participation in annual well-child visits may not substantially change the cost-effectiveness, perhaps due to the low depression prevalence among symptom-free patients. We demonstrated that universal routine screening could be more cost-effective if coupled with increased treatment access to care or effectiveness. For example, treatment effectiveness can be improved by integrating primary and mental care services delivery or implementing collaborative care models.^37,68,69^ As telemedicine use trends upwards for behavioral health care,^70^ universal routine screening combined with treatment may be increasingly cost-effective if adolescents do not require caregiver time costs in psychotherapy (via telemedicine). By conducting a cost-effectiveness model and robust uncertainty analyses, we can pinpoint opportunities for future value of information analysis or evidence collection to inform screening effectiveness. In our study, we found that collecting additional data on treatment effectiveness by disaggregated age, sex, and racial or ethnic groups and clarifying the adolescent-specific depression utilities across varying severities may offer insights into screening cost-effectiveness.

Previous cost-effectiveness analyses compared adolescent depression treatment options, but to our knowledge none examined screening specifically.^71,72,73^ Two cost-effectiveness studies evaluated universal depression screening in adults but not adolescents. Valenstein et al^74^ found that universal annual depression screening in adult primary care yielded $352 891 per QALY gained over the lifetime from the societal perspective. Jiao et al^75^ found that universal single-time, 2-staged screening (using PHQ-2 and PHQ-9) with collaborative care treatment yielded $2225 per QALY gained among urban adults. Both ICERs are adjusted for inflation. Our analysis found that universal annual screening in adolescents yielded $57 795 per QALY gained compared with usual care and $66 822 per QALY gained compared with single-time screening over 10 years from the societal perspective. We expected that universal annual screening in adolescents would be more cost-effective than in adults because of their higher depression prevalence and if they underwent screening routinely for longer periods.

### Limitations

This study had limitations. This study relied on calibration targets from National Survey on Drug Use and Health, which applies the Center for Epidemiologic Studies Depression Scale screener.^76^ Still, the restricted National Health and Nutrition Examination Survey,^77,78^ which applies the more popular PHQ-9, might provide alternative inputs.^79^ We did not assess treatment-resistant patients or patients who switch treatments, which are common circumstances.^7,50,57^ The 10-year horizon ended at age 22 years, resulting in conservative results. Using a longer time horizon may capture additional health benefits, reducing depression symptoms early and lessening the associated long-term health risks, like cardiovascular disease.^80^ Family caregiver spillover effects meaningfully affect QALYs^81,82^ but were not incorporated in our model given the lack of standardized methods for their consideration.^22^ We used adult health utilities as to our knowledge there are no reported adolescent depression-related utilities.^83,84^ We did not include other potentially important societal costs, like presenteeism, future non–health-related consumption, or school-based effects. Screening would likely be even more cost-effective if additional productivity losses were included.^22,75^ There is a need to collect disaggregated depression-related data among other marginalized groups to facilitate the development of programs or policies for reducing mental health disparities.

## Conclusions

In this economic evaluation, universal annual depression screening for adolescents and young adults in primary care was cost-effective compared with commonly used willingness-to-pay thresholds. The findings highlight the importance of improved and coordinated care along the adolescent mental care delivery continuum to better identify high-value interventions, including enhanced family access to telemedicine behavioral health, lower treatment costs, or improved treatment efficiency and effectiveness. Disaggregated data are needed to accurately model adolescent depression and care access experiences.^85^ It is imperative that health care systems enhance their capacity to detect, diagnose, and treat major depression early to improve adolescent health and well-being.

## Notes

Supplement 1. eTable 1. Impact inventory eTable 2. CHEERS 2022 checklist eFigure 1. State-transition depression sub-model for universal routine screening of major depression for adolescents in primary care settings eFigure 2. One-way tornado analyses comparing a single-time screening vs. usual care and annual screening vs. single-time screening with ICERs from the limited societal perspective, discounted eTable 3. One-way sensitivity analyses comparing single-time screening vs. usual care and annual screening vs. single-time screening, ICERs from the limited societal perspective, discounted eTable 4. Projected total and incremental costs and quality-adjusted life years and ICERs per 1,000 U.S. adolescents from ages 12-22 years by disaggregated sex and race or ethnicity combinations, discounted eTable 5. Additional scenario analyses; discounted annually by 3%, base-case ICER comparing single-time screening vs. usual care=USD$44,483/QALY, base-case ICER comparing annual screening vs. single-time screening=USD$66,822/QALY, from the limited societal perspective

Supplement 2. Data sharing statement
