# Errors in Results and Table 1

**DOI:** 10.1001/jamahealthforum.2025.2603

**Published:** 2025-06-13

In the Original Investigation titled “Cost-Effectiveness of Universal Routine Depression Screening for Adolescents in Primary Care,”^1^ published online on May 2, 2025, the phrase “was associated with” was erroneously included in a sentence in the Results section that should instead read “Reducing the time horizon from 10 years to 3 years increased ICERs.” Additionally, in Table 1, the superscript s beside the fifth to last row (“Relative risk of suicidality”) should be a superscript t, the superscript q beside the second to last row (“Relative risk of suicidality”) should be a superscript r, and the superscript t in the last row (“Probability of discontinuing treatment”) should be a superscript s. This article was corrected online.
