# Supplementary material for: Cost-Effectiveness of Universal Routine Depression Screening for Adolescents in Primary Care
Source: JAMA Health Forum. 2025 May 2;6(5):e250711. doi: 10.1001/jamahealthforum.2025.0711 (PMC12048853; doi:10.1001/jamahealthforum.2025.0711)
Supplement: Supplement 1. — eTable 1. Impact inventory eTable 2. CHEERS 2022 checklist eFigure 1. State-transition depression sub-model for universal routine screening of major depression for adolescents in primary care settings eFigure 2. One-way tornado analyses comparing a single-time screening vs. usual care and annual screening vs. single-time screening with ICERs from the limited societal perspective, discounted eTable 3. One-way sensitivity analyses comparing single-time screening vs. usual care and annual screening vs. single-time screening, ICERs from the limited societal perspective, discounted eTable 4. Projected total and incremental costs and quality-adjusted life years and ICERs per 1,000 U.S. adolescents from ages 12-22 years by disaggregated sex and race or ethnicity combinations, discounted eTable 5. Additional scenario analyses; discounted annually by 3%, base-case ICER comparing single-time screening vs. usual care=USD$44,483/QALY, base-case ICER comparing annual screening vs. single-time screening=USD$66,822/QALY, from the limited societal perspective [file jamahealthforum-e250711-s001.pdf]

Supplemental Online Content

Doan TT, Hutton DW, Wright DR, Prosser LA. Cost-effectiveness of universal routine depression screening for adolescents in primary care. *JAMA Health Forum*. 2025;6(5):e250711. doi:10.1001/jamahealthforum.2025.0711

eTable 1. Impact inventory

eTable 2. CHEERS 2022 checklist

eFigure 1. State-transition depression sub-model for universal routine screening of major depression for adolescents in primary care settings

eFigure 2. One-way tornado analyses comparing a single-time screening vs. usual care and annual screening vs. single-time screening with ICERs from the limited societal perspective, discounted

eTable 3. One-way sensitivity analyses comparing single-time screening vs. usual care and annual screening vs. single-time screening, ICERs from the limited societal perspective, discounted

eTable 4. Projected total and incremental costs and quality-adjusted life years and ICERs per 1,000 U.S. adolescents from ages 12-22 years by disaggregated sex and race or ethnicity combinations, discounted

eTable 5. Additional scenario analyses; discounted annually by 3%, base-case ICER comparing single-time screening vs. usual care=USD$44,483/QALY, base-case ICER comparing annual screening vs. single-time screening=USD$66,822/QALY, from the limited societal perspective

This supplemental material has been provided by the authors to give readers additional information about their work.

© 2025 Doan TT et al. *JAMA Health Forum*.

eTable 1. Impact Inventory

Type of impact	Healthcare sector perspective	Limited societal perspective
**Formal healthcare sector**		
Health outcomes		
Longevity effects	☐	☐
This analysis had a limited time horizon, from adolescence into early adulthood.		
Health related quality of life effects	☑	☑
Universal depression screening and enhanced treatment are intended to improve quality of life		
Other health effects	☑	☑
Secondary outcomes were evaluated: depression-free days.		
Medical costs		
Medical costs paid by third-party payers.	☑	☑
Included costs of physician visits, depression screening, and depression treatment.		
Medical costs paid for out-of-pocket	☐	☐
Out-of-pocket costs were not included		
Future related medical costs	☐	☐
Future costs were not included		
Future unrelated medical costs	☐	☐
Future costs were not included		
**Informal healthcare sector**		
Patient time costs	☐	☑
Time costs related to physician visits and screening were added to the limited societal perspective		
Unpaid caregiver costs	☐	☐
Not applicable to the condition.		
Transportation costs	☐	☐
Not included.		
**Non-healthcare sectors**		
Productivity losses	☐	☐
Not included.		
Consumption	☐	☐
Not included.		
Cost of social services	☐	☐
Not included.		
**Cost of crimes related to intervention**	☐	☐
Not included.		

© 2025 Doan TT et al. *JAMA Health Forum*.

eTable 2. CHEERS 2022 Checklist (Husereau et al. 2022)

Topic	No.	Item	Location where item is reported
**Title**			
	1	Identify the study as an economic evaluation and specify the interventions being compared.	Title, Page 1
**Abstract**			
	2	Provide a structured summary that highlights context, key methods, results, and alternative analyses.	Abstract, Page 2
**Introduction**			
**Background and objectives**	3	Give the context for the study, the study question, and its practical relevance for decision making in policy or practice.	Introduction
**Methods**			
**Health economic analysis plan**	4	Indicate whether a health economic analysis plan was developed and where available.	Methods, Sub-sections Base-case analysis and Uncertainty Analyses
**Study population**	5	Describe characteristics of the study population (such as age range, demographics, socioeconomic, or clinical characteristics).	Methods, Sub-section Approach
**Setting and location**	6	Provide relevant contextual information that may influence findings.	Introduction; Methods Sub-section Event probabilities)
**Comparators**	7	Describe the interventions or strategies being compared and why chosen.	Methods, Sub-section Interventions
**Perspective**	8	State the perspective(s) adopted by the study and why chosen.	Methods, Sub-section Base-case analysis
**Time horizon**	9	State the time horizon for the study and why appropriate.	Methods, Sub-sections Approach
**Discount rate**	10	Report the discount rate(s) and reason chosen.	Methods, Sub-section Base-case analysis
**Selection of outcomes**	11	Describe what outcomes were used as the measure(s) of benefit(s) and harm(s).	Methods, Sub-section Base-case analysis
**Measurement of outcomes**	12	Describe how outcomes used to capture benefit(s) and harm(s) were measured.	Methods, Sub-section Costs and QALYs; Table 1
**Valuation of outcomes**	13	Describe the population and methods used to measure and value outcomes.	Methods, Sub-sections Approach, Costs, and QALYs; Table 1
**Measurement and valuation of resources and costs**	14	Describe how costs were valued.	Methods, Sub-section Costs; Table 1
**Currency, price date, and conversion**	15	Report the dates of the estimated resource quantities and unit costs, plus the currency and year of conversion.	Methods, Sub-section Costs; Table 1
**Rationale and description of model**	16	If modelling is used, describe in detail and why used. Report if the model is publicly available and where it can be accessed.	Methods, Sub-section Model structure
**Analytics and assumptions**	17	Describe any methods for analysing or statistically transforming data, any extrapolation methods, and approaches for validating any model used.	Methods, Sub-section Event probabilities; Figure A1 describing Depression Sub-model

© 2025 Doan TT et al. *JAMA Health Forum*.

Topic	No.	Item	Location where item is reported
**Characterising heterogeneity**	18	Describe any methods used for estimating how the results of the study vary for subgroups.	Methods, Sub-section Uncertainty Analysis
**Characterising distributional effects**	19	Describe how impacts are distributed across different individuals or adjustments made to reflect priority populations.	Not reported
**Characterising uncertainty**	20	Describe methods to characterise any sources of uncertainty in the analysis.	Methods, Sub-section Uncertainty Analysis; Supplementary Appendix
**Approach to engagement with patients and others affected by the study**	21	Describe any approaches to engage patients or service recipients, the general public, communities, or stakeholders (such as clinicians or payers) in the design of the study.	Methods, Sub-section Model inputs and data sources using Expert Opinion
**Results**			
**Study parameters**	22	Report all analytic inputs (such as values, ranges, references) including uncertainty or distributional assumptions.	Methods, Table 1
**Summary of main results**	23	Report the mean values for the main categories of costs and outcomes of interest and summarise them in the most appropriate overall measure.	Results
**Effect of uncertainty**	24	Describe how uncertainty about analytic judgments, inputs, or projections affect findings. Report the effect of choice of discount rate and time horizon, if applicable.	Results & Supplementary Appendix
**Effect of engagement with patients and others affected by the study**	25	Report on any difference patient/service recipient, general public, community, or stakeholder involvement made to the approach or findings of the study	Not reported
**Discussion**			
**Study findings, limitations, generalisability, and current knowledge**	26	Report key findings, limitations, ethical or equity considerations not captured, and how these could affect patients, policy, or practice.	Discussion
**Other relevant information**			
**Source of funding**	27	Describe how the study was funded and any role of the funder in the identification, design, conduct, and reporting of the analysis	Title page
**Conflicts of interest**	28	Report authors conflicts of interest according to journal or International Committee of Medical Journal Editors requirements.	Title page

From: Husereau D, Drummond M, Augustovski F, et al. Consolidated Health Economic Evaluation Reporting Standards 2022 (CHEERS 2022) Explanation and Elaboration: A Report of the ISPOR CHEERS II Good Practices Task Force. Value Health 2022;25. doi:10.1016/j.jval.2021.10.008.

© 2025 Doan TT et al. *JAMA Health Forum*.

eFigure 1. State-transition depression sub-model for universal routine screening of major depression for adolescents in primary care settings.

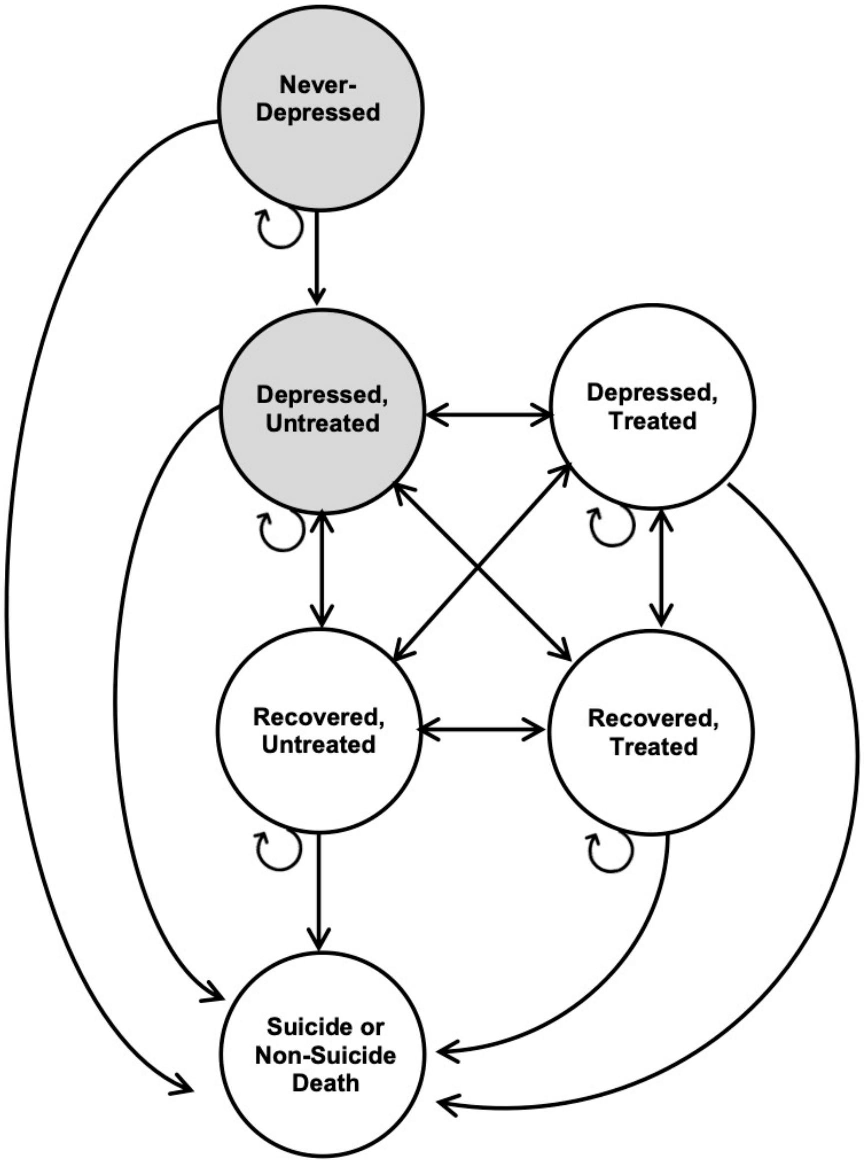

Circles indicated health states. Shaded circles indicated the initial states that the cohort could begin in. Arrows indicated direction of transitions.

© 2025 Doan TT et al. *JAMA Health Forum*.

eFigure 2. One-way tornado analyses comparing a single-time screening vs. usual care and annual screening vs. single-time screening with ICERs from the limited societal perspective, discounted.

A)

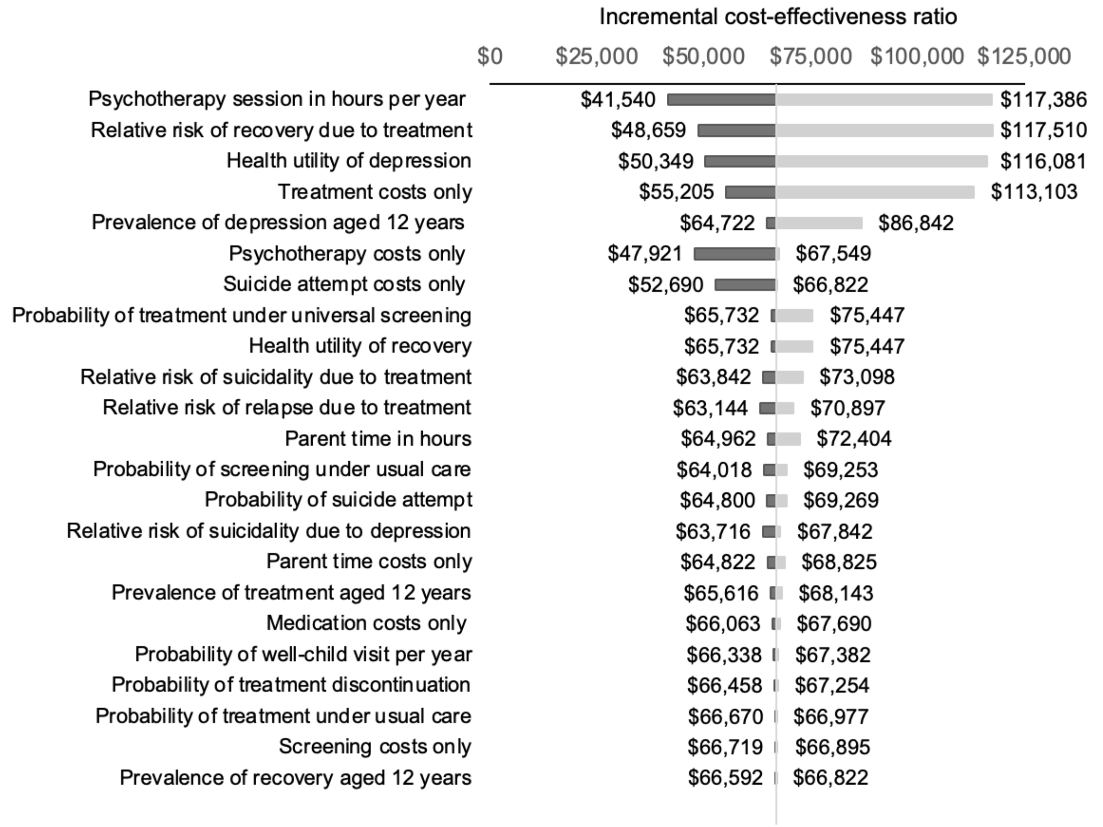

B)

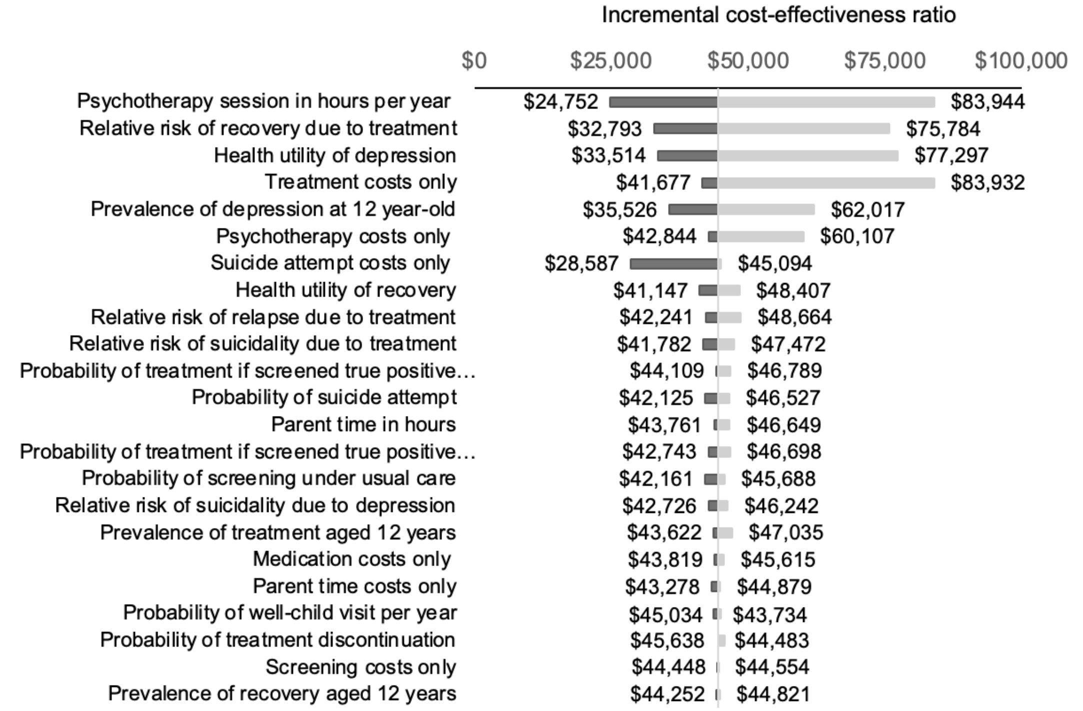

Abbreviations: ICER=incremental cost-effectiveness ratio; QALYs=quality-adjusted life years

© 2025 Doan TT et al. *JAMA Health Forum*.

eTable 3. One-way sensitivity analyses comparing single-time screening vs. usual care and annual screening vs. single-time screening, ICERs (USD$2023/QALY) from the limited societal perspective, discounted.

Variables	Universal single-time screening vs. usual care		Universal annual screening vs. single-time screening	
	ICER, sensitivity ranges	% change from base-case, sensitivity ranges	ICER, sensitivity ranges	% change from base-case, sensitivity ranges
	(lower bound sensitivity range values–upper bound sensitivity range values)			
	Base-case ICER ($/QALY) = USD$44,483		Base-case ICER ($/QALY) = USD$66,822	
Prevalence of depression aged 12 years	$35,526–$62,017	-20.1%; 39.4%	$64,722–$86,842	-3.1%; 30.0%
Prevalence of recovery aged 12 years	$44,252–$44,821	-0.5%; 0.8%	$66,592–$66,822	-0.3%; 0.0%
Prevalence of treatment aged 12 years	$43,622–$47,035	-1.9%; 5.7%	$65,616–$68,143	-1.8%; 2.0%
Probability of well-child visit per year	$43,734–$45,034	-1.7%; 1.2%	$66,338–$67,382	-0.7%; 0.8%
Probability of screening under usual care	$42,161–$45,688	-5.2%; 2.7%	$64,018–$69,253	-4.2%; 3.6%
Probability of treatment if screened true positive under usual care	$42,743–$46,698	-3.9%; 5.0%	$66,670–$66,977	-0.2%; 0.2%
Probability of treatment if screened true positive under universal screening	$44,109–$46,789	-0.8%; 5.2%	$65,732-$75,447	-1.6%; 12.9%
Probability of treatment discontinuation	$44,483–$45,638	0.0%; 2.6%	$66,458–$67,254	-0.5%; 0.6%
Probability of suicide attempt	$42,125–$46,527	-5.3%; 4.6%	$64,800–$69,269	-3.0%; 3.7%
Screening costs only	$44,448–$44,554	-0.1%; 0.2%	$66,719–$66,895	-0.2%; 0.1%
PhQ-9 Sensitivity	$43,599- $45,374	-2.0%; 2.0%	$65,971; $68,272	-1.3%; 2.2%
PhQ-9 Specificity	$44,472-$44,515	0.0%; 0.1%	$66,817-$66,833	0.0%; 0.0%
Treatment costs only	$41,677–$83,932	-6.3%; 88.7%	$55,205–$113,103	-17.4%; 69.3%
Psychotherapy costs only	$42,844–$60,107	-3.7%; 35.1%	$47,921–$67,549	-28.3%; 1.1%
Medication costs only	$43,819–$45,615	-1.5%; 2.5%	$66,063–$67,690	-1.1%; 1.3%
Suicide attempt costs only	$28,587–$45,094	-35.7%; 1.4%	$52,690–$66,822	-21.1%; 0.0%
Parent time costs only	$43,278–$44,879	-2.7%; 0.9%	$64,822–$68,825	-3.0%; 3.0%
Parent time in hours (bundled screening, visits, and travel)	$43,761–$46,649	-1.6%; 4.9%	$64,962–$72,404	-2.8%; 8.4%
Psychotherapy session in hours per year	$24,752–$83,944	-44.4%; 88.7%	$41,540–$117,386	-37.8%; 75.7%
Health utility of depression	$33,514–$77,297	-24.7%; 73.8%	$50,349–$116,081	-24.7%; 73.7%
Health utility of recovery	$41,147–$48,407	-7.5%; 8.8%	$65,732–$75,447	-1.6%; 12.9%
Relative risk of relapse due to treatment	$42,241–$48,664	-5.0%; 9.4%	$63,144–$70,897	-5.5%; 6.1%
Relative risk of recovery due to treatment	$32,793–$75,784	-26.3%; 70.4%	$48,659–$117,510	-27.2%; 75.9%
Relative risk of suicidality due to treatment	$41,782–$47,472	-6.1%; 6.7%	$63,842–$73,098	-4.5%; 9.4%
Relative risk of suicidality due to depression	$42,726–$46,242	-3.9%; 4.0%	$63,716–$67,842	-4.6%; 1.5%

Abbreviations: ICER=incremental cost-effectiveness ratio; QALYs=quality-adjusted life years

© 2025 Doan TT et al. *JAMA Health Forum*.

eTable 4. Projected total and incremental costs and quality-adjusted life years and ICERs (USD$2023/QALY) per 1,000 U.S. adolescents from ages 12-22 years by disaggregated sex and race or ethnicity combinations, discounted.

A)

	**Female, American Indian/Alaska Native**					**Male, American Indian/Alaska Native**				
Strategy	Total costs (in thousands)	Incr. costs (in thousands)	Total QALYs	Incr. QALYs gained	$/QALY gained	Total costs (in thousands)	Incr. costs (in thousands)	Total QALYs	Incr. QALYs gained	$/QALY gained
Usual care[a]	$10,510	-	7,562	-	-	$8,349	-	8,106	-	-
Single-time screening	$11,598	$1,088	7,576	14.4	$75,435	$8,796	$447	8,107	0.6	Dominated
Biennial screening	$12,958	$1,360	7,594	17.7	Dominated	$9,350	$554	8,107	0.7	Dominated
Annual Screening	$14,597	$1,639	7,615	21.4	$76,711	$10,147	$797	8,109	1.1	$734,023

B)

	**Female, Asian, Hawaiian or Other Pacific Islander**					**Male, Asian, Hawaiian or Other Pacific Islander**				
Strategy	Total costs (in thousands)	Incr. costs (in thousands)	Total QALYs	Incr. QALYs gained	$/QALY gained	Total costs (in thousands)	Incr. costs (in thousands)	Total QALYs	Incr. QALYs gained	$/QALY gained
Usual care[a]	$10,259	-	7,549	-	-	$8,194	-	8,166	-	-
Single-time screening	$11,487	$1,228	7,558	8.4	$145,672	$8,616	$423	8,171	4.5	$93,834
Biennial screening	$13,152	$1,665	7,568	10.1	Dominated	$9,155	$539	8,176	5.1	$105,311
Annual Screening	$15,503	$2,350	7,583	14.8	$161,297	$9,982	$828	8,182	6.0	$137,948

© 2025 Doan TT et al. *JAMA Health Forum*.

C)

	Female, Black/African American					Male, Black/African American				
Strategy	Total costs (in thousands)	Incr. costs (in thousands)	Total QALYs	Incr. QALYs gained	$/QALY gained	Total costs (in thousands)	Incr. costs (in thousands)	Total QALYs	Incr. QALYs gained	$/QALY gained
Usual care[a]	$9,952	-	7,729	-	-	$8,065	-	8,189	-	-
Single-time screening	$11,026	$1,074	7,753	23.6	$45,599	$8,472	$406	8,191	1.8	$212,479
Biennial screening	$12,075	$1,048	7,768	15.6	Dominated	$8,861	$389	8,193	1.9	Dominated
Annual Screening	$13,571	$1,496	7,795	26.5	$60,549	$9,547	$686	8,194	1.8	$388,287

D)

	Female, Hispanic/Latina/Spanish					Male, Hispanic/Latina/Spanish				
Strategy	Total costs (in thousands)	Incr. costs (in thousands)	Total QALYs	Incr. QALYs gained	$/QALY gained	Total costs (in thousands)	Incr. costs (in thousands)	Total QALYs	Incr. QALYs gained	$/QALY gained
Usual care[a]	$11,178	-	7,457	-	-	$9,575	-	7,806	-	-
Single-time screening	$12,672	$1,494	7,488	30.6	$48,771	$10,432	$857	7,819	12.8	$67,041
Biennial screening	$14,115	$1,443	7,514	26.4	$54,572	$11,419	$987	7,833	14.7	$67,191
Annual Screening	$16,032	$1,917	7,549	34.6	$55,404	$12,741	$1,322	7,850	16.5	$80,156

© 2025 Doan TT et al. *JAMA Health Forum*.

E)

	Female, White					Male, White				
Strategy	Total costs (in thousands)	Incr. costs (in thousands)	Total QALYs	Incr. QALYs gained	$/QALY gained	Total costs (in thousands)	Incr. costs (in thousands)	Total QALYs	Incr. QALYs gained	$/QALY gained
Usual care[a]	$10,846	-	7,534	-	-	$8,798	-	7,976	-	-
Single-time screening	$12,234	$1,388	7,566	32.1	$43,311	$9,504	$706	7,992	16.1	$43,741
Biennial screening	$13,279	$1,044	7,574	7.8	Dominated	$10,156	$652	7,995	2.9	Dominated
Annual Screening	$14,816	$1,537	7,600	26.5	$75,287	$11,282	$1,126	8,012	16.5	$91,298

F)

	Female, Multiracial or Other Race or Ethnicity					Male, Multiracial or Other Race or Ethnicity				
Strategy	Total costs (in thousands)	Incr. costs (in thousands)	Total QALYs	Incr. QALYs gained	$/QALY gained	Total costs (in thousands)	Incr. costs (in thousands)	Total QALYs	Incr. QALYs gained	$/QALY gained
Usual care[a]	$12,869	-	7,051	-	-	$10,082	-	7,625	-	-
Single-time screening	$15,079	$2,210	7,099	48.1	$45,924	$11,696	$1,614	7,638	13.6	$118,979
Biennial screening	$16,571	$1,492	7,126	26.4	Dominated	$12,505	$809	7,637	-1.6	Dominated
Annual Screening	$18,804	$2,233	7,178	52.6	$47,128	$14,515	$2,819	7,649	11.0	$257,189

[a]Usual care was defined as a 20% annual screening rate and 33% treatment initiation rate.

Abbreviations: ICER=incremental cost-effectiveness ratio; incr=incremental; QALYs=quality-adjusted life years

© 2025 Doan TT et al. *JAMA Health Forum*.

eTable 5. Additional scenario analyses; discounted annually by 3%, base-case ICER comparing single-time screening vs. usual care=USD$44,483/QALY, base-case ICER comparing annual screening vs. single-time screening=USD$66,822/QALY, from the limited societal perspective.

Scenario	Scenario ICER	% change from base-case ICER
**Psychotherapy-only**		
Single-time screening vs. usual care	$188,470	323.7%
Annual screening vs. single-time screening	$340,172	409.1%
**Medication-only**		
Single-time screening vs. usual care	$21,447	-51.8%
Annual screening vs. single-time screening	$50,150	-24.9%
**Status-quo well-child visit and status-quo treatment rates**[a]		
Single-time screening vs. usual care	$49,275	10.8%
Annual screening vs. single-time screening	$82,184	23.0%
**Enhanced well-child visit and status-quo treatment rates**[a]		
Single-time screening vs. usual care	$48,915	10.0%
Annual screening vs. single-time screening	$84,377	26.3%
**Enhanced well-child visit and enhanced treatment rates**[a]		
Single-time screening vs. usual care	$42,551	-4.3%
Annual screening vs. single-time screening	$69,953	4.7%
**Added parent time costs for transporting children to in-person psychotherapy**[b]		
Single-time screening vs. usual care	$58,095	30.6%
Annual screening vs. single-time screening	$84,264	26.1%
**Added adolescent time costs to clinician exams and in-person psychotherapy**[c]		
Single-time screening vs. usual care	$46,893	5.4%
Annual screening vs. single-time screening	$70,512	5.5%
**No treatment discontinuation**[d]		
Single-time screening vs. usual care	$45,638	2.6%
Annual screening vs. single-time screening	$66,592	-0.3%
**Treatment re-initiation after discontinuation**[e]		
Single-time screening vs. usual care	$45,292	1.8%
Annual screening vs. single-time screening	$66,414	-0.6%
**Discount rate = 1.5%**		
Single-time screening vs. usual care	$43,726	-1.7%
Annual screening vs. single-time screening	$65,319	-2.2%
**Discount rate = 5%**		

© 2025 Doan TT et al. *JAMA Health Forum*.

Single-time screening vs. usual care	$45,515	2.3%
Annual screening vs. single-time screening	$68,869	3.1%
**Time horizon = 12 to 15 years of age**		
Single-time screening vs. usual care	$57,806	30.0%
Annual screening vs. single-time screening	$157,591	135.8%
**Time horizon = 12 to 18 years of age**		
Single-time screening vs. usual care	$49,870	12.1%
Annual screening vs. single-time screening	$98,282	47.1%

[a] This scenario contrasted with the base-case universal screening options, which assumed status-quo well-child visit and enhanced treatment rates. Enhanced well-child visit rate was set to 0.9.

[b]Assumed time costs of parents were 1.5 hours, in which 1 hour was allocated for psychotherapy and 0.5 hour for transportation.

[a]Assumed hourly wage of adolescents was $7.25 (the federal minimum wage in 2023) was added to clinical exams and psychotherapy.

[d] This scenario contrasted with the base-case, which assumed treatment can be discontinued after the first year of treatment initiation.

[e] This scenario contrasted with the base-case, which assumed treatment did not start again after treatment discontinuation.

Abbreviations: ICER=incremental cost-effectiveness ratio; QALYs=quality-adjusted life years

© 2025 Doan TT et al. *JAMA Health Forum*.

eReferences

1. Doan TT, Hutton DW, Wright DR, Prosser LA. Estimating Transition Probabilities for Modeling Major Depression in Adolescents by Sex and Race or Ethnicity Combinations in the USA. *Appl Health Econ Health Policy*. 2024:1-16.
2. Harris KM, Udry JR. National Longitudinal Study of Adolescent to Adult Health (Add Health), 1994-2008. *UNC Carolina Popul Cent Proj*. 2014:1994-2008. doi:10.1002/jbm.820281214
3. Riera-Serra P, Navarra-Ventura G, Castro A, et al. Clinical predictors of suicidal ideation, suicide attempts and suicide death in depressive disorder: a systematic review and meta-analysis. *Eur Arch Psychiatry Clin Neurosci*. 2023. doi:10.1007/s00406-023-01716-5
4. Substance Abuse and Mental Health Services Administration. National Survey on Drug Use and Health. 2019;(November):(online). https://nsduhweb.rti.org. Accessed August 21, 2021.
5. Rodgers CRR, Flores MW, Bassey O, Augenblick JM, Cook BL. Racial/Ethnic Disparity Trends in Children's Mental Health Care Access and Expenditures From 2010-2017: Disparities Remain Despite Sweeping Policy Reform. *J Am Acad Child Adolesc Psychiatry*. 2022;61(7):915-925. doi:10.1016/j.jaac.2021.09.420
6. Flores MW, Sharp A, Carson NJ, Cook BL. Estimates of major depressive disorder and treatment among adolescents by race and ethnicity. *JAMA Pediatr*. 2023;177(11):1215-1223.
7. Merikangas KR, He J, Burstein M, et al. Service utilization for lifetime mental disorders in US adolescents: results of the National Comorbidity Survey–Adolescent Supplement (NCS-A). *J Am Acad Child Adolesc Psychiatry*. 2011;50(1):32-45.
8. Centers for Disease Control and Prevention (CDC). CDC WONDER. doi:10.5860/choice.45-6201
9. Centers for Medicare and Medicaid Services (CMS). Physician Fee Schedule Look-Up Tool. https://www.cms.gov/medicare/payment/fee-schedules/physician/lookup-tool. Published 2024. Accessed April 3, 2024.
10. Washington State Department of Health. Youth Suicide FAQs. 2010. Washington State Department of Health. https://doh.wa.gov/you-and-your-family/injury-and-violence-prevention/suicide-prevention/youth-suicide-prevention/youth-suicide-faqs. Published 2011. Accessed June 3, 2022.
11. Perkins R. *Alaska Suicide Hospitalizations 2001 - 2002*.; 2005. https://health.alaska.gov/SuicidePrevention/Documents/pdfs_sspc/SuicideHospitalizations.pdf.
12. Shepard DS, Gurewich D, Lwin AK, Reed GA, Silverman MM. Suicide and Suicidal Attempts in the United States: Costs and Policy Implications. *Suicide Life Threat Behav*. 2016;46(3):352-362. doi:10.1111/sltb.12225
13. U.S. Department of Veterans Affairs. Office of Procurement, Acquisition and Logistics. Federal Supply Schedule. Pharmaceutical Prices. 2023. https://www.va.gov/opal/nac/fss/pharmPrices.asp. Accessed April 3, 2024.
14. U.S. Bureau of Labor Statistics. Occupational Employment Statistics. https://www.bls.gov/ces/. Published 2023. Accessed April 3, 2024.
15. CMS. Medicaid and Chip Scorecard 2023. Medicaid.gov. https://www.medicaid.gov/state-overviews/scorecard/welcome. Published 2024. Accessed September 5, 2022.
16. Sekhar DL, Ba DM, Liu G, Kraschnewski JL. Major Depressive Disorder Screening Remains Low Even Among Privately Insured Adolescents. *J Pediatr*. 2019;204:203-207. doi:10.1016/j.jpeds.2018.07.086
17. Bose J, Zeno R, Warren B, Sinnott LT, Fitzgerald EA. Implementation of universal adolescent depression screening: quality improvement outcomes. *J Pediatr Heal Care*. 2021;35(3):270-277.
18. Lewandowski RE, O'Connor B, Bertagnolli A, et al. Screening for and Diagnosis of Depression Among Adolescents in a Large Health Maintenance Organization. *Psychiatr Serv*. 2016;67(6):636-641. doi:10.1176/appi.ps.201400465
19. Zenlea IS, Milliren CE, Mednick L, Rhodes ET. Depression screening in adolescents in the United States: A national study of ambulatory office-based practice. *Acad Pediatr*. 2014;14(2):186-191. doi:10.1016/j.acap.2013.11.006
20. MN Community Measurement. Minnesota Health Care Quality Report 2019. https://mncmsecure.org/website/Reports/Community Reports/Health Care Quality Report/2020 HCQR Chartbook FINAL.pdf. Published 2021. Accessed August 3, 2022.
21. Richardson LP, McCauley E, Grossman DC, et al. Evaluation of the Patient Health Questionnaire-9 Item for Detecting Major Depression Among Adolescents. *Pediatrics*. 2010;126(6):1117-1123. doi:10.1542/peds.2010-0852
22. Gaylor EM. Suicidal thoughts and behaviors among high school students—Youth Risk Behavior Survey, United States, 2021. *MMWR Suppl*. 2023;72.
23. Ward ZJ, Barrett JL, Cradock AL, et al. Childhood Obesity Intervention Cost-Effectiveness Study (CHOICES)

© 2025 Doan TT et al. *JAMA Health Forum*.

Microsimulation Model Technical Documentation: Details on Model Parameters.
24. Mann R, Gilbody S, Richards D. Putting the "Q" in depression QALYs: A comparison of utility measurement using EQ-5D and SF-6D health related quality of life measures. *Soc Psychiatry Psychiatr Epidemiol*. 2009;44(7):569-578. doi:10.1007/s00127-008-0463-5
25. Sapin C, Fantino B, Nowicki M-L, Kind P. Usefulness of EQ-5D in assessing health status in primary care patients with major depressive disorder. *Health Qual Life Outcomes*. 2004;2(1):1-8.
26. Domino ME, Burns BJ, Silva SG, et al. Cost-effectiveness of treatments for adolescent depression: Results from TADS. *Am J Psychiatry*. 2008;165(5):588-596. doi:10.1176/appi.ajp.2008.07101610
27. Emslie GJ, Kennard BD, Mayes TL, et al. Fluoxetine versus placebo in preventing relapse of major depression in children and adolescents. *Am J Psychiatry*. 2008;165(4):459-467.
28. Lynch FL, Dickerson JF, Clarke GN, et al. Cost-Effectiveness of Preventing Depression Among At-Risk Youths: Postintervention and 2-Year Follow-Up. *Psychiatr Serv*. 2019;70(4):279-286. doi:10.1176/appi.ps.201800144
29. Kennard B, Silva S, Vitiello B, et al. Remission and residual symptoms after short-term treatment in the Treatment of Adolescents with Depression Study (TADS). *J Am Acad Child Adolesc Psychiatry*. 2006;45(12):1404-1411.
30. March J, Silva S, Petrycki S, et al. Fluoxetine, cognitive-behavioral therapy, and their combination for adolescents with depression: Treatment for Adolescents With Depression Study (TADS) randomized controlled trial. *Jama*. 2004;292(7):807-820.
31. Vitiello B, Silva SG, Rohde P, et al. Suicidal events in the treatment for adolescents with depression study (TADS). *J Clin Psychiatry*. 2009;70(5):741-747. doi:10.4088/JCP.08m04607
32. March JS, Silva S, Petrycki S, et al. The Treatment for Adolescents With Depression Study (TADS): long-term effectiveness and safety outcomes. *Arch Gen Psychiatry*. 2007;64(10):1132-1143.

© 2025 Doan TT et al. *JAMA Health Forum*.
