# Supplementary material for: Cost-Effectiveness of Universal Routine Depression Screening for Adolescents in Primary Care
Source: JAMA Health Forum. 2025 May 2;6(5):e250711. doi: 10.1001/jamahealthforum.2025.0711 (PMC12048853; doi:10.1001/jamahealthforum.2025.0711)
Supplement: Supplement 2. — Data sharing statement [file jamahealthforum-e250711-s002.pdf]

Data Sharing Statement

Doan. Cost-Effectiveness of Universal Routine Depression Screening for Adolescents in Primary Care. *JAMA Health Forum*. Published May 02, 2025.
doi:10.1001/jamahealthforum.2025.0711

Data
Data available: No

Additional Information
Explanation for why data not available: The model inputs and sources used in the cost-effectiveness model are made available in Table 1.
